# BNP as a New Biomarker of Cardiac Thyroid Hormone Function

**DOI:** 10.3389/fphys.2020.00729

**Published:** 2020-07-09

**Authors:** Kaihao Wang, Kaie Ojamaa, Abigail Samuels, Nimra Gilani, Kuo Zhang, Shimin An, Youhua Zhang, Yi-Da Tang, Bardia Askari, Anthony Martin Gerdes

**Affiliations:** ^1^Department of Biomedical Sciences, New York Institute of Technology College of Osteopathic Medicine, Old Westbury, NY, United States; ^2^Department of Cardiology, State Key Laboratory of Cardiovascular Disease, Fuwai Hospital, National Center for Cardiovascular Diseases, Chinese Academy of Medical Sciences and Peking Union Medical College, Beijing, China

**Keywords:** BNP, thyroid hormones, T3, hypothyroidism, heart failure, gene expression

## Abstract

**Background:**

Cardiac re-expression of fetal genes in patients with heart failure (HF) suggests the presence of low cardiac tissue thyroid hormone (TH) function. However, serum concentrations of T3 and T4 are often normal or subclinically low, necessitating an alternative serum biomarker for low cardiac TH function to guide treatment of these patients. The clinical literature suggests that serum Brain Natriuretic Peptide (BNP) levels are inversely associated with serum triiodo-L-thyronine (T3) levels. The objective of this study was to investigate BNP as a potential serum biomarker for TH function in the heart.

**Methods:**

Two animal models of thyroid hormone deficiency: (1) 8-weeks of propyl thiouracil-induced hypothyroidism (Hypo) in adult female rats were subsequently treated with oral T3 (10 μg/kg/d) for 3, 6, or 14 days; (2) HF induced by coronary artery ligation (myocardial infarction, MI) in adult female rats was treated daily with low dose oral T3 (5 μg/kg/d) for 8 or 16 wks.

**Results:**

Six days of T3 treatment of Hypo rats normalized most cardiac functional parameters. Serum levels of BNP increased 5-fold in Hypo rats, while T3 treatment normalized BNP by day 14, showing a significant inverse relationship between serum BNP and free or total T3 concentrations. Myocardial BNP mRNA was increased 2.5-fold in Hypo rats and its expression was decreased to normal values by 14 days of T3 treatment. Measurements of hemodynamic function showed significant dysfunction in MI rats after 16 weeks, with serum BNP increased by 4.5-fold and serum free and total T3 decreased significantly. Treatment with T3 decreased serum BNP while increasing total T3 indicating an inverse correlation between these two biologic factors (*r*^2^ = 0.676, *p* < 0.001). Myocardial BNP mRNA was increased 5-fold in MI rats which was significantly decreased by T3 over 8 to 16 week treatment periods.

**Conclusions:**

Results from the two models of TH dysfunction confirmed an inverse relationship between tissue and serum T3 and BNP, such that the reduction in serum BNP could potentially be utilized to monitor efficacy and dosing of T3 treatment. Thus, serum BNP may serve as a reliable biomarker for cardiac TH function.

## Introduction

In recent years, numerous clinical studies have shown a significant association between low thyroid hormone (TH) function and worsening cardiac function, and increased mortality in heart failure (HF; Tseng et al., 2012; Mitchell et al., 2013; Chuang et al., 2014; Wang et al., 2015; Brozaitiene et al., 2016; Gil-Cayuela et al., 2017, 2018; She et al., 2018; Sato et al., 2019). Increased mortality has been reported in patients with HF within the lower half of the normal reference range for T3 compared to the upper half (Sato et al., 2019). Nonetheless, low TH function in patients with heart disease typically goes untreated largely due to many unanswered questions related to treatment optimization, fear of inducing arrhythmias, and identification of the patient cohort that may obtain the greatest benefit from treatment.

In response to these concerns, a serum biomarker reflecting cardiac tissue TH function is needed. Ideally, the serum biomarker should be secreted by the myocardium into the circulation when cardiac tissue TH function is below normal, and then be suppressed when TH function is normalized by treatment. A search of the pre-clinical and clinical literature suggests that the fetal gene B-type or Brain Natriuretic Peptide (BNP) may serve this purpose. Two small clinical trials using T3 treatment of HF patients supports this hypothesis. Amin et al. (2015) demonstrated improvement of LV function in T3 treated HF patients who had a significant reduction in serum BNP levels. Holmager et al. (2015) found that when BNP levels were unchanged in HF patients treated with T3, no improvement in cardiac function was recorded, suggesting inadequate dosing. These examples indicate the potential value of a serum biomarker that is already in widespread use and would directly correlate with TH function in the heart tissue itself. Our extensive experimental studies and those of other investigators have indicated that serum T3 and T4 do not directly correlate with cardiac tissue hormone levels and, therefore, another serum biomarker is required to guide treatment (Escobar del Rey et al., 1989; Escobar-Morreale et al., 2005; Liu et al., 2008; Weltman et al., 2013). We have published data that show a correlation of measured cardiac tissue T3 concentration on contractile function that in turn is due to the effects on expression of T3 responsive genes including calcium regulatory and myofilament proteins (Weltman et al., 2013). Those data show that tissue T3 associates directly with cardiac function as measured by LV rate of pressure development (+dP/dt and -dP/dt), and that tissue T3 content determines αMHC expression which in turn correlates with contractile function.

The objective in the present study was to use expression of the T3-responsive α-MHC gene as a surrogate of tissue T3 action and correlate this with tissue BNP expression and with serum BNP and T3 levels. The goal is to provide evidence of the utility of serum BNP as a biomarker of TH action in the heart. These data are timely in light of an on-going recently funded NIH clinical study aimed to develop oral L-T3 therapy for HF (NCT04111536).

## Materials and Methods

### Animal Models and Treatment Protocols

All animals were treated in accordance with the United States Public Health Service “Guide for the Care and Use of Laboratory Animals” (Guidelines for the Care and Use of Laboratory Animals, 2011, 8th edition), and study protocols were approved by the Institutional Animal Care and Use Committee of the New York Institute of Technology.

#### Hypothyroid Model

Adult female Sprague Dawley rats (220–240*g*; Envigo, Indianapolis, IN, United States) were randomly assigned to either hypothyroid or euthyroid groups. Hypothyroidism was established by 8 weeks of treatment with 0.025% 6-n-propyl-2-thiouracil (PTU; Sigma, St Louis, MO, United States) dissolved in drinking water as previously described (Tang et al., 2005). After 8 weeks of PTU treatment, rats were randomly assigned to continued PTU treatment or to receive PTU plus T3 (SigmaAldrich) that was added to the PTU-containing drinking water for an additional 3, 6, or 14 days. Assuming no impairment in T3 intestinal absorption, this oral T3 concentration corresponds to 10 μg/kg/d. Untreated age-matched rats served as euthyroid controls (EU). Rats were divided into the following groups (*n* = number of animals): EU (*n* = 8); PTU (*n* = 9); PTU + 3d T3 (*n* = 6); PTU + 6d T3 (*n* = 6), and PTU + 14d T3 (*n* = 10). Number of animals/group for some reported parameters vary as indicated in Figure legends. PTU was administered for the entire duration of the experiment to avoid endogenous TH production. Water consumption was measured twice per week to adjust T3 concentration based on body weight and water consumed. All animals were kept on a 12-h light, 12-h dark cycle, and food and water were provided *ad libitum*.

#### Heart Failure Model

Adult female Sprague-Dawley rats approximately 3 months of age (220–250*g*; Envigo, Indianapolis, IN, United States) were subjected to left anterior descending coronary artery ligation to produce myocardial infarction (MI) or underwent sham surgery without occlusion of the coronary vessel as previously published (Rajagopalan et al., 2016). The day after surgery, surviving MI animals were randomized to receive T3 or vehicle in drinking water for 8 or 16 wks in separate studies, each with appropriate 8 or 16 week sham and vehicle treated groups. T3 was provided in drinking water at 5 μg/kg/d as reported previously (Rajagopalan et al., 2016). MI + Veh (vehicle) rats received ethanol/glycerol formulation in drinking water equivalent to that in the treatment groups. The rats had access to food and water *ad libitum*. The number of animals per group are indicated in the figure legends, with some differences in numbers depending on the parameter measured.

### Echocardiographic Measurements

At the end of the treatment period, echocardiography was performed using a GE Vivid 7 Dimension System (GE Vingmed Ultrasound, Horten, Norway) coupled with a M12L linear (Matrix) array ultrasound transducer probe (5–13 MHz). Rats were lightly anesthetized with isoflurane (1.5%), and a parasternal short-axis view was obtained in B-Mode and recorded in M-mode. Body temperature was maintained with a heating pad for all echocardiographic and hemodynamic measurements. Myocardial wall movement was traced over three to five cardiac cycles to measure left ventricular (LV) anterior and posterior wall thickness in end-diastole and -systole, and LV diastolic and systolic internal diameters, LV fractional shortening as described previously (Zhang et al., 2018).

### Cardiac Hemodynamic Measurements

Immediately following echocardiographic recordings, under continued isoflurane anesthesia, right carotid artery catheterization was performed using a 1.9F pressure catheter (Transonic SciSense, Canada), and the tip of the catheter was advanced into the LV as previously published (Zhang et al., 2018). The following data were recorded over 15–20 min: heart rate (HR), LV systolic pressure (LVSP), LV end-diastolic pressure (LVEDP), positive and negative change in LV pressure over time (±dP/dt), and Tau (time constant for isovolumic relaxation).

### Serum Thyroid Hormone and BNP Assays

Following functional measurements in closed chest animals, a left thoracotomy exposed the heart, and blood was obtained from the right ventricular cavity. Blood was left to clot at room temperature for 30 min and then centrifuged at 1800 rpm for 15 min at 4°C. Serum was collected, aliquoted, and stored at −20°C until it was analyzed. Concentration of the bioactive 45 amino acid BNP molecule was determined by a quantitative assay based on the competitive enzyme immunoassay principle (RayBiotech Life, Peachtree Corners, GA, United States). Note: after testing several commercially available kits for serum BNP types in rats, this was the most reliable and consistent in our hands. Analysis of free T3, total T3, and total T4 used enzyme-linked immunosorbent assay kits according to the manufacturers’ protocols (Monobind Inc., Lake Forest, CA, United States). In the 8-wks MI study, we assayed serum THs and refroze for subsequent BNP assays. Unfortunately, we discovered that refreezing sera caused significant degradation of BNP in this assay.

### Real-Time Quantitative PCR

Frozen left ventricular tissues were pulverized and ∼50 mg samples were homogenized in QIAzol lysis reagent. Total RNA was extracted using RNeasy Mini spin columns (Qiagen, Germantown, MD, United States) and RNA concentration was measured by absorbance at 260 nm using a spectrophotometer (QuickDrop, Molecular Devices). Using anchored-oligo(dT)_18_ and random hexamer primers (Transcriptor First Strand cDNA Synthesis Kit, Roche Diagnostics Corp, Indianapolis, IN, United States), cDNA was reverse transcribed from 0.5–1 μg RNA. Real-time PCR (StepOnePlus, Applied Biosystems ThermoFisher) using SYBR Green technology (RT^2^ SYBR Green ROX qPCR, Qiagen) was used to amplify Myh6 (α-MHC), Myh7 (β-MHC), and Nppb (BNP) expressed genes with specific primers designed and verified by Qiagen. GAPDH was used as the house-keeping gene for normalization of amplified PCR products. Data analysis used the ΔΔCt method and results for each gene are expressed as fold changes relative to the mean value of the sham or EU groups.

### Statistical Analysis

Data are presented as mean ± SD and group means were compared using one-way ANOVA with Tukey’s *post hoc* multiple-group comparisons. Spearman’s correlation analysis was used to determine the correlation between values. All data analyses passed tests for normality distribution and equal variance using GraphPad Prism v7.0 statistical software (GraphPad Software, Inc., San Diego, CA, United States). Statistical significance was accepted at *p* < 0.05.

## Results

### Hypothyroid Model

#### Morphometric Changes

As previously reported with Hypo induced by either PTU or surgical thyroidectomy, weight gain is depressed (Liu et al., 2008; Weltman et al., 2013). Consequently, body weight was significantly less in PTU treated rats compared with the EU group which continued to gain weight (209 ± 16 *g* vs 256 ± 26 *g*, respectively; Supplementary Table). Differences in LV weight between PTU and EU reflected the combined effects of these changes in body weight and thyroid status (461 ± 22 mg vs 616 ± 66 mg). After 14 days of T3 treatment, heart weight was normalized but body weight was not yet fully restored.

#### Echocardiography Data

Cardiac function declined after PTU treatment with ejection fraction (EF) values significantly decreased compared to EU (EF, 70 ± 5 vs 82 ± 1%; Supplementary Table). These changes were normalized within the 3–14 day period of T3 treatment. Echocardiography showed significant changes in measures of wall thickness and LV chamber diameters in both diastole and systole (Supplementary Table). All these parameters were significantly improved by the third day of T3 treatment, demonstrating restoration of normal cardiac dimensions and function. LV internal diameter in systole (LVIDs) was increased in PTU hearts (4.6 ± 0.4 mm vs 3.5 ± 0.3 mm, PTU vs EU, respectively, *p* < 0.05) and by 3 days of T3 treatment, LVIDs decreased significantly to 3.6 ± 0.6 mm. Posterior wall thickness in systole (PWTs) was significantly decreased to 1.7 ± 0.0 mm in PTU hearts vs 2.1 ± 0.1 mm in EU, and normalized to 2.0 ± 0.1 mm after 3 days of T3 treatment.

#### Hemodynamic Assessment

**FIGURE 1:**
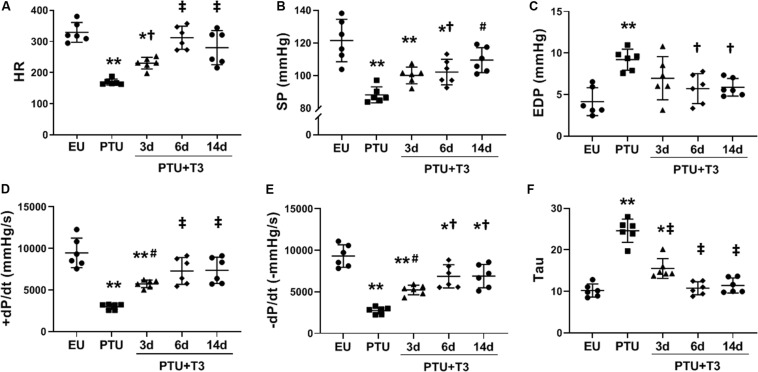
LV hemodynamics in the hypothyroid model. Scatter dot plots show horizontal lines representing group mean SD; each dot represents a measured value from an individual animal. **(A)** heart rate (HR); **(B)** LV maximum systolic pressure (SP); **(C)** LV end diastolic pressure (EDP); **(D)** positive (+) and **(E)** negative (-) change in pressure over time (±dP/dt); and **(F)** time constant for isovolumic relaxation (Tau). Animals/group: EU, *n* = 6; PTU, *n* = 6; 3d T3, *n* = 6; 6d T3, *n* = 6; 14d T3, and *n* = 6. Statistical analysis used one-way ANOVA with Tukey’s *post hoc* multiple-group comparisons. *p < 0.01 vs EU, **p < 0.001 vs EU, ^†^*p* < 0.05 vs PTU, ^#^*p* < 0.01 vs PTU, and ^‡^*p* < 0.001 vs PTU.

Significant bradycardia was apparent in the PTU rats and HR was increased significantly by day 3 of T3 treatment, and further normalized by day 6 (Figure 1A). At this T3 dose, HR at 14 days of T3 treatment was not different than EU controls. LV pressure measurements recorded in lightly anesthetized animals showed a significant decrease in systolic pressure (SP) and a significant increase in end diastolic pressure (EDP) in the PTU group compared with EU and these parameters were normalized with T3 treatment within 6–14 days (Figures 1B,C). T3 treatment significantly improved rate of pressure development (+dP/dt) by 3 days and normalized this parameter within 6 days of treatment (Figure 1D). During diastole, –dP/dt and the Tau constant showed significant impairment in the PTU group and these measurements were improved with T3 treatment within 3–14 days (Figures 1E,F).

#### Serum TH and BNP Levels

**FIGURE 2:**
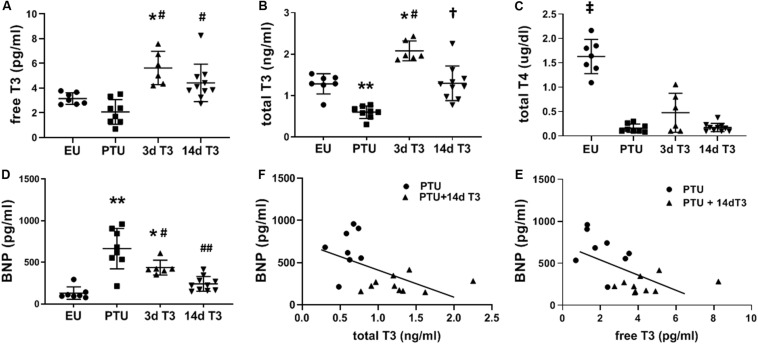
Serum thyroid hormone and BNP concentrations in the hypothyroid model. Scatter dot plots show horizontal lines representing group mean ± SD; each dot represents the value from an individual animal. **(A)** free triiodo-L-thyronine (T3); **(B)** total T3; **(C)** total L-thyroxine (T4); and **(D)** serum BNP. Animals/group: EU, *n* = 7; PTU, *n* = 8; 3d T3, *n* = 6; 14d T3, and *n* = 10. Statistical analysis used one-way ANOVA with Tukey’s *post hoc* multiple-group comparisons. *p < 0.01 vs EU, **p < 0.001 vs EU, ^#^*p* < 0.01 vs PTU, ^##^*p* < 0.001 vs PTU, ^†^*p* < 0.001 vs PTU and 3d T3, and ^‡^*p* < 0.0001 vs all groups. **(E)** correlation between serum BNP and total T3 in 14d T3 treated rats; Spearman correlation coefficient *R*^2^ = 0.296, *p* = 0.020; and **(F)** correlation between serum BNP and free T3 in 14d T3 treated rats; Spearman correlation coefficient *R*^2^ = 0.342, *p* = 0.011. Best fit linear regression line is indicated.

Serum total T3 concentrations were significantly lower in the PTU group compared with the EU, with serum free T3 trending lower (Figures 2A,B). Serum total T4 was significantly decreased in PTU rats compared to EU and remained depressed in the PTU rats treated with T3 as would be expected due to the inhibitory effects of PTU on thyroperoxidase and on peripheral tissue conversion of T4 to T3 by inhibiting 5’-deiodinase (Figure 2C).

Compared to EU rats, serum BNP was significantly increased by 5-fold in the PTU group (Figure 2D). T3 treatment significantly lowered serum BNP by day 3, and by day 14 of treatment BNP was not different from EU values. There were significant inverse correlations between serum BNP and serum total and free T3 in rats of both PTU and PTU + 14 days T3 treated groups (Figures 2E,F), with Spearman correlation coefficients of *r*^2^ = 0.296, *p* = 0.02 and *r*^2^ = 0.342, *p* = 0.01, respectively.

#### T3 Regulated Cardiac Genes

**FIGURE 3:**
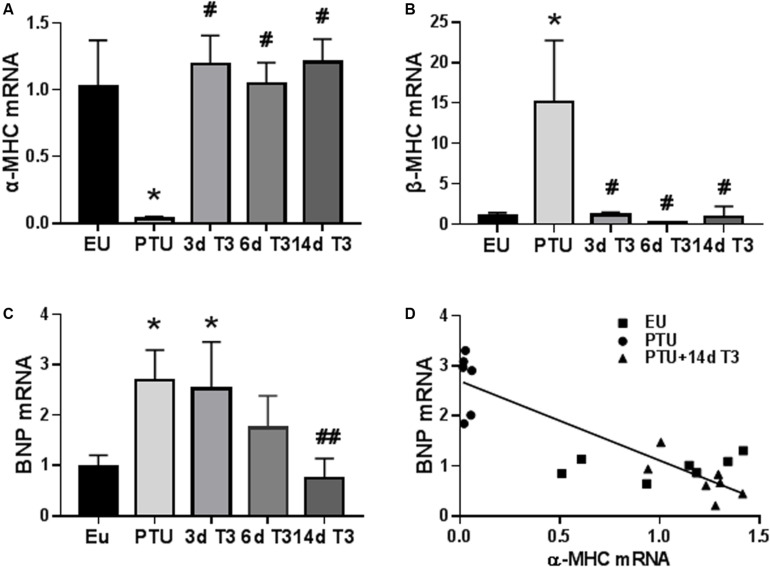
Expression of cardiac genes in the hypothyroid model. Bar graphs show group mean ± SD and represents the fold change compared to EU group. **(A)** myosin heavy chain α isoform (α-MHC); **(B)** MHC β isoform (β-MHC); and **(C)** B-type natriuretic peptide (BNP). Animals/group: EU, *n* = 7; PTU, *n* = 6; 3d T3, *n* = 6; 6d T3, *n* = 6; and 14d T3, *n* = 7. Statistical analysis used one-way ANOVA with Tukey’s *post hoc* multiple-group comparisons. *p < 0.01 vs EU, ^#^*p* < 0.01 vs PTU. ^##^*p* < 0.05 vs PTU, 3d and 6d T3. **(D)** correlation between BNP and α-MHC mRNA in 14d T3 treated rats; Spearman correlation coefficient *r*^2^ = 0.476, *p* < 0.001. Linear regression line is shown.

The expression of the T3-responsive cardiac myosin heavy chain genes, α-MHC, and β-MHC, which are involved in active force generation, were significantly altered in the PTU group (Figures 3A,B). Within 3 days of T3 treatment, expression of these two genes was completely normalized. BNP mRNA content in the PTU rat hearts was significantly increased 2.5-fold (Figure 3C), consistent with the increase in serum BNP concentrations (Figure 2D). In contrast to the MHC genes, expression of BNP was decreased more slowly by T3 treatment, showing reduced expression by day 6, and reaching EU values by day 14 of treatment. A significant inverse correlation (*r*^2^ = 0.476, *p* < 0.001) exists between gene expression of BNP and α-MHC suggesting negative T3-responsiveness of the BNP gene (Figure 3D) and a potential association with tissue T3 content.

### MI Model With 16 Weeks T3 Treatment

#### LV Hemodynamics

**FIGURE 4:**
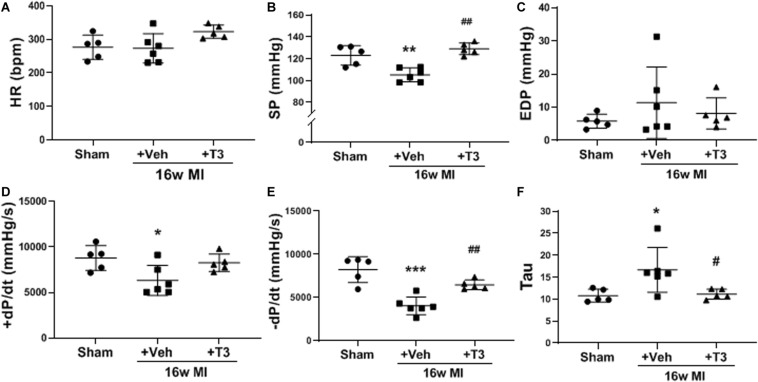
LV hemodynamics in the MI model, 16 weeks MI plus vehicle (Veh) or T3 treatment. **(A)** heart rate (HR); **(B)** LV maximum systolic pressure (SP); **(C)** LV end diastolic pressure; **(D)** positive (+) and **(E)** negative (−) change in pressure over time (+/−dP/dt); and **(F)** time constant for isovolumic relaxation (Tau). Each dot represents values from one animal. One-way ANOVA with Tukey’s *post hoc* analysis. *p < 0.05 vs Sham, **p < 0.01 vs Sham, ***p < 0.0001 vs Sham, ^#^*p* < 0.05 vs MI + Veh, and ^##^*p* < 0.01 vs MI + Veh.

Significant LV dysfunction was observed in most hemodynamic measurements recorded at 16 weeks post-MI including maximum LV systolic pressure developed (SP) and ±dP/dt (Figures 4B–F) suggesting HF. The low-dose T3 treatment for the 16 week period improved these outcomes significantly without an increase in HR response (Figure 4A).

#### Serum BNP and THs

**FIGURE 5:**
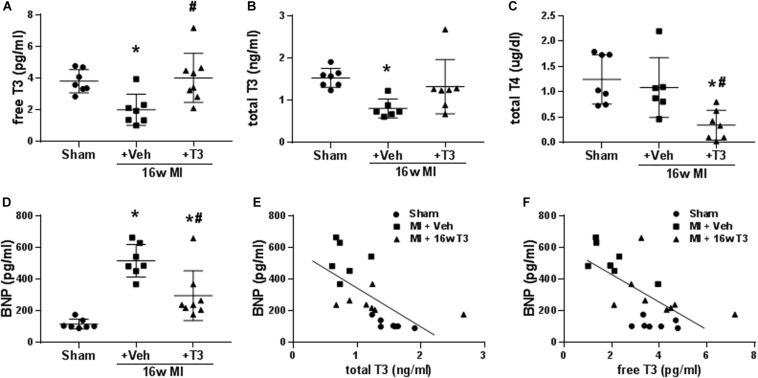
Serum thyroid hormone and BNP concentrations in the MI model, 16 wk MI plus vehicle (Veh) or T3 treatment. **(A)** free T3; **(B)** total T3; **(C)** total T4; and **(D)** serum BNP. Animals/group: Sham, *n* = 7; MI + Veh, *n* = 6–7; and MI+16-week T3, *n* = 7–8. One-way ANOVA with Tukey’s *post hoc* multiple-group comparisons; *p < 0.05 vs Sham, ^#^*p* < 0.05 vs MI + Veh. **(E)** correlation between serum BNP and total T3 of all rats from the study groups; Spearman correlation coefficient *r*^2^ = 0.676, *p* < 0.001; **(F)** correlation between serum BNP and free T3; Spearman correlation coefficient *r*^2^ = 0.412, *p* < 0.01. Linear regression line is indicated.

Both free and total T3 were significantly lower in the MI rats after 16 weeks with normal serum T4 indicating low T3 syndrome (Figures 5A–C). The dose of T3 administered normalized serum T3 concentrations, while total T4 was significantly reduced as would be expected from negative feedback regulation of the hypothalamic-pituitary-thyroid axis (Figures 5A–C). Serum BNP in these rats was significantly elevated by 4.5-fold compared to sham animals supporting incidence of HF (Figure 5D). T3 treatment reduced serum BNP levels significantly compared to vehicle-treated MI rats, while BNP was still higher than that in sham rats. Importantly, serum BNP showed significant inverse correlations with both total T3 and free T3 concentrations (*r*^2^ = 0.676, *p* < 0.001 and *r*^2^ = 0.412, *p* < 0.01, respectively; Figures 5E,F).

### MI Model With 8 Weeks T3 Treatment

#### Morphometric Changes and LV Function

Eight weeks after MI, heart weight and heart to body weight ratios were significantly (*p* < 0.0001) increased compared to the sham animals (ratios: 3.36 ± 0.14, 4.49 ± 0.88, and 4.72 ± 0.40; sham, MI + Veh, and MI+T3, respectively), indicating hypertrophy.

**FIGURE 6:**
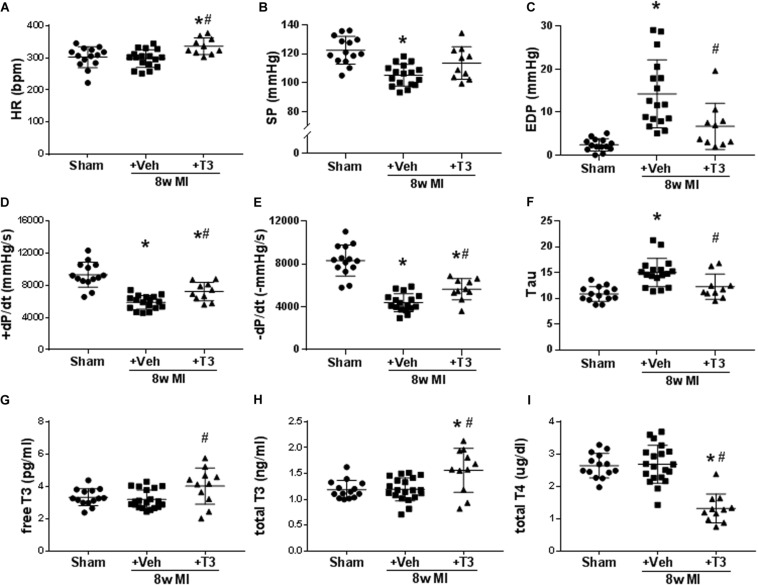
LV hemodynamic measurements and serum thyroid hormone concentrations in 8 wk MI plus vehicle (Veh) or T3 treatment. Scatter dot plots are as described in the legend to Figure 1. **(A)** HR; **(B)** SP; **(C)** EDP; **(D)** +dP/dt; **(E)** -dP/dt; **(F)** Tau; **(G)** serum free T3; **(H)** total T3; and **(I)** total T4. Animals/group: Sham, *n* = 14; MI + Veh, *n* = 17–20; and MI+8-week T3, *n* = 11. Statistical analysis used one-way ANOVA with Tukey’s *post hoc* multiple-group comparisons. *p < 0.05 vs Sham, ^#^*p* < 0.05 vs MI + Veh.

MI resulted in significant reductions of rates of LV pressure development and relaxation (±dp/dt), with increased Tau constant and reduced maximum systolic pressure development (Figures 6B–F). LV EDP was elevated significantly in MI (14.3 ± 7.7 vs 2.4 ± 1.4 mmHg) and this was reduced after 8 weeks of T3 treatment (6.7 ± 5.1 mmHg; Figure 6C). HR was not altered in the MI + Veh group but exhibited a ∼10% increase with T3 treatment (Figure 6A).

#### Serum TH Levels

At the end of the 8 wks study period, no differences were observed in serum free T3 or total T3 concentrations between MI + Veh and sham groups (Figures 6G,H). However, small but significant increases were measured with T3-treatment, while total T4 was decreased as predicted (Figure 6I). We have previously reported in animal models of HF that the administered dose of T3 preserves cardiac tissue T3 levels while maintaining serum THs within the normal range (Weltman et al., 2014, 2015).

#### Gene Expression Studies

**FIGURE 7:**
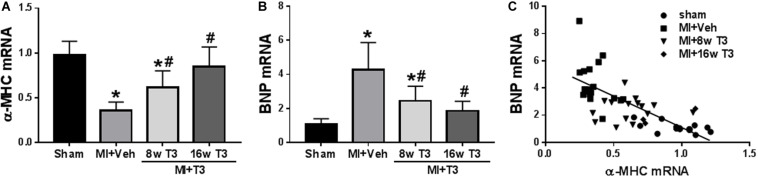
Expression of cardiac genes in the MI model (8 wk T3 and 16 wk T3 groups). Bar graphs show group mean ± SD and represents the fold change compared to the Sham group. **(A)** α-MHC; **(B)** BNP. Statistical analysis used one-way ANOVA with Tukey’s *post hoc* multiple-group comparisons. *p < 0.05 vs Sham, ^#^*p* < 0.05 vs MI + Veh. **(C)** correlation between BNP and α-MHC mRNAs showing individual animal values in all study groups; Spearman correlation coefficient *r*^2^ = 0.667, *p* < 0.001. MI + Veh includes rats from 8- and 16-week groups. Linear regression line indicated.

Changes in expression of α-MHC were measured at 8 and 16 weeks after MI, while T3 treatment largely reversed these changes (Figure 7A). BNP mRNA increased significantly by 5-fold in the MI rats, and this was normalized after 16 weeks of low dose T3 treatment (Figure 7B). The T3-responsiveness of the BNP and α-MHC genes showed a significant inverse relationship with a correlation coefficient of *r*^2^ = 0.667 (*p* < 0.001; Figure 7C).

## Discussion

### Major Findings

Cardiac tissue from rats with HF or hypothyroidism showed increased BNP and fetal gene expression. In both cases, T3 treatment improved cardiac function, reversed cardiac fetal gene expression, downregulated cardiac BNP expression, and decreased serum BNP. These results indicate that serum BNP reduction after T3 treatment confirms reversal of cardiac fetal gene expression, normalization of cardiac TH function, and improved LV function. This close inverse relationship between T3 and BNP suggests that BNP may be a reliable serum biomarker for low cardiac tissue thyroid function in HF and heart diseases leading to this syndrome. Results from the MI study suggest that T3 doses may be precisely and safely titrated with resulting downregulation of serum BNP reflecting concurrent reversal of cardiac fetal gene expression and downregulation of BNP mRNA, changes associated with improvement of cardiac function.

### Low TH Function and HF

Many clinical studies in recent years have shown worsening outcomes and increased mortality in HF patients with borderline low TH conditions such as Low T3 Syndrome and subclinical hypothyroidism (Tseng et al., 2012; Mitchell et al., 2013; Chuang et al., 2014; Wang et al., 2015; Sato et al., 2019). In fact, a recent study reported higher cardiac and all-cause mortality in HF patients in the lower half of the normal reference range for serum T3 (Sato et al., 2019). While two recent studies showed low cardiac tissue T3 levels in samples from HF patients (Gil-Cayuela et al., 2017, 2018), we were unable to confirm the reliability of the ELISA assay employed in those studies. We are not aware of any other human cardiac tissue T3 data using the Mass Spectrometry (MS) method validated by Zucchi’s group in Italy (Saba et al., 2014), which to our knowledge is the only group in the world doing this assay. Nonetheless, low cardiac tissue T3 in human HF is likely since animal studies of heart diseases have consistently shown a reduction in tissue T3 levels measured by MS even when serum T3 levels are normal (Pol et al., 2011; Weltman et al., 2014, 2015). We have conducted four studies examining rats with graded levels of TH function (hypothyroidism to hyperthyroidism; Liu et al., 2008; Weltman et al., 2013), diabetic cardiomyopathy (Weltman et al., 2014), and hypertension (Weltman et al., 2015), with cardiac tissue T3 samples analyzed by Zucchi’s group. Pol et al. (2011) also collaborated with Zucchi to examine cardiac tissue T3 levels in mice with MI. In all cases, changes in cardiac tissue T3 levels, TH response genes, and LV function were intimately linked. In particular, changes in α-MHC/β-MHC ratio serves as a reliable indicator of cardiac tissue TH activity and T3 levels. It is also clear from these studies that serum T3 levels do not reflect cardiac tissue T3 levels in many cases, particularly in borderline low TH conditions. This underscores a fundamental problem in treating this condition and highlights the critical need for a serum biomarker tracking with cardiac tissue TH activity.

### T3, BNP, α-MHC Relationship

BNP and NT-proBNP have been used routinely to determine the severity of human HF. Many clinical studies have shown an inverse relationship between serum BNP and serum T3 levels in patients who were not hyperthyroid (Bunevicius et al., 2006; Pinelli et al., 2007; Mayer et al., 2008; Pfister et al., 2010; Du et al., 2012; Brozaitiene et al., 2016; She et al., 2018; Sato et al., 2019). Based on this observation, we explored the utility of BNP as a measure of cardiac tissue TH function using animal models of hypothyroidism and MI. Upregulation of α-MHC, a sensitive and proven marker of T3 gene activation within cardiac myocytes, was used as a measure of tissue T3 content and thus was confirmation of a positive T3 response.

### Hypothyroidism Increases BNP

The PTU study showed that cardiac tissue BNP mRNA and serum BNP were increased in hypothyroidism, along with changes in other known T3-responsive genes including upregulation of β-MHC and downregulation of α-MHC, changes typical of HF. T3 quickly reversed expression of these cardiac-specific fetal genes within 3 days of treatment, with reduction of serum BNP also noted at that time point. The high levels of serum BNP as well as myocardial BNP mRNA in the PTU animals were reduced slowly over the T3 treatment period until normal levels were attained by day 14, suggesting that the BNP expression is T3-responsive, but the effect may not be direct and may involve other regulatory factors including changes in hemodynamic load. We have used expression of the α-MHC gene as a measure of tissue T3 content, and in this regard, as tissue α-MHC increased, BNP mRNA decreased as supported by the significant inverse correlation of these two expressed genes.

Notably, the 2.5-fold increase in myocardial BNP mRNA was reflected in the 5-fold increase in serum BNP concentrations. Importantly, serum BNP concentrations were reduced slowly during the 14-day treatment period, similar to BNP mRNA. Thus, as T3 treatment normalized gene expression and BNP production and secretion, cardiac contractile function was similarly improved over time. Thus, we posit that serum BNP is a valuable biomarker of T3 function in the heart.

As suggested from studies by Gabrielle Escobar (Escobar-Morreale et al., 2005) and our previous studies (Liu et al., 2008; Weltman et al., 2013), monotherapy with either T3 or T4 requires high normal serum doses to fully restore cardiac tissue TH function in Hypo rats. Escobar also noted that serum euthyroidism with combined T3 and T4 therapy achieved restoration of cardiac T3 levels. The high normal T3 dose selected for the Hypo rats in the current study reflects this situation and precisely duplicates our previous results (Chen et al., 2013; Weltman et al., 2014, 2015). There is no evidence that the Hypo + T3 rats in the current study were hyperthyroid based on the submitted data. Fortunately, multiple studies from our lab have clearly demonstrated that, unlike the situation with primary Hypo, Hypo secondary to heart diseases does not require a higher dose of T3 for restoration of cardiac tissue TH function (Weltman et al., 2014, 2015; Zhang et al., 2018). This is fortunate from a clinical treatment standpoint.

### BNP and T3 Treatment of HF

Serum T3 levels were significantly reduced in 16-week but not 8-week MI rats suggesting progressive deterioration of TH function in failing hearts. T3 treatment normalized serum free T3 and total T3 in 16-week MI rats. While T3 treatment led to a significant increase in serum free T3 and total T3 in 8-week MI rats, it should be noted that values were within normal ranges with no evidence of induction of hyperthyroidism. MI-induced HF led to increased β-MHC and BNP, and reduced α-MHC mRNA levels, and T3 treatment reversed these changes with improvements in various parameters of cardiac function. The significant inverse relationship between serum BNP and T3 suggests that serum BNP may be a reliable and sensitive biomarker for cardiac tissue TH signaling. Furthermore, reduction of serum BNP after T3 treatment indicates restoration of myocardial TH function as evidenced by parallel reversal of fetal gene expression.

Brain Natriuretic Peptide is a small cardiac natriuretic peptide hormone first identified in porcine brain tissue (Sudoh et al., 1988). The human BNP gene is located on chromosome 1 and encodes the prohormone pro-BNP (Hunt et al., 1995). In the circulation, biologically active BNP is separated from the n-terminal part of the 76 amino acid prohormone NT-proBNP (Hunt et al., 1995). BNP is produced mainly in ventricular myocytes and increased stretch of cardiomyocytes has been shown to trigger the expression of BNP (Blaauw et al., 2010). In heart diseases, there is increased expression of BNP, and other genes that are typically expressed at higher levels during fetal growth (Man et al., 2018). Many studies have shown that elevated BNP is an independent predictor of mortality and other cardiac outcomes in patients with heart diseases (Balion et al., 2006; Di Angelantonio et al., 2009). BNP is used as a diagnostic, management, and prognostic tool for HF (Thygesen et al., 2012; Daubert et al., 2019). An unconfirmed report by Liang et al. (2003) described a thyroid response element (TRE) on the BNP gene and T3 induced expression of BNP in neonatal cultured myocytes. But BNP is generally increased during fetal growth when TH levels are low, arguing against T3 activation of BNP (Cameron and Ellmers, 2003).

After a careful survey of the clinical literature, a strong inverse correlation between serum BNP and T3 in cardiac patients was noted in most studies (Bunevicius et al., 2006; Mayer et al., 2008; Pfister et al., 2010; Du et al., 2012; Brozaitiene et al., 2016; She et al., 2018; Sato et al., 2019). Indeed, the clinical literature spurred us to conduct the animal studies reported herein to confirm this inverse relationship. This consistent inverse correlation between serum BNP and T3 also suggested that BNP may be a potential biomarker for low cardiac tissue TH function. While BNP is also increased in hyperthyroidism (Ozmen et al., 2007), BNP values were reported to be normal in hyperthyroid patients with normal cardiac function (Wei et al., 2005), suggesting that stretch activation rather than elevated TH function was responsible. Certainly, the use of BNP as a marker of negative regulation by TH would be irrelevant in hyperthyroid patients who would never be considered for TH treatment. An inverse relationship between BNP and T3 has also been reported in non-cardiac patients with low TH function (Pinelli et al., 2007). Hajje et al. (2014) showed that PTU treated rats had increased serum and cardiac tissue BNP, increased β-MHC, and reduced α-MHC. As expected, these values were normal 6 weeks after restoration of TH function. They did not investigate temporal downregulation of BNP by T3, mention the possibility of negative regulation of BNP by T3, or investigate the potential use of BNP as a biomarker for low cardiac tissue TH function. Data submitted here and the preponderance of human data within the normal TH reference range and below offers strong support for the diagnostic use of BNP in this manner.

She et al. (2018) reported BNP values ∼3 times higher in MI patients just below the normal reference range for total T3 and free T3 vs those within the normal reference range. Sato et al. (2019) examined the clinical profiles of HF patients within the normal reference range of serum total T3 and free T3. Patients in the lower half of the normal reference range for free T3 and total T3 had significantly higher cardiac and all-cause mortality than those in the upper half of the normal T3 reference range. BNP values for patients in the lower half were more than double those in the upper half of the normal reference range. Finally, published T3 treatment studies of HF patients also support our hypothesis. Pingitore et al. (2008) reported a reduction in serum BNP in HF patients treated for 3 days with T3. The two clinical studies cited earlier showed LV functional improvement in T3 treated HF patients with reduced BNP (Amin et al., 2015) but no improvement when T3 treatment did not reduce BNP compared to placebo (Holmager et al., 2015). Results of these clinical studies and our animal experiments here suggest that serum BNP values could be used to guide low dose T3 treatment of patients in HF patients with borderline low TH function and possibly those in the lower half of the T3 reference range. Importantly, evidence indicates that reduction of serum BNP confirms restoration of cardiac TH signaling. We think this report should increase confidence and precision for TH treatment of patients with borderline low TH conditions using BNP as a guide for gradual titration of T3 treatment. Changes in serum BNP should also be predictive of the efficacy of T4 or combination T3/T4 treatment of cardiac patients.

### Additional Clinical Implications

Translation of our findings may result in much broader benefits than may be initially apparent. A growing body of evidence suggests that impaired microvascular blood flow may be a common contributor to most heart diseases leading to HF. For instance, impaired microvascular blood flow has been found in ischemic heart disease (non-infarcted areas; Mygind et al., 2016), idiopathic dilated cardiomyopathy (van den Heuvel et al., 2000; Roura and Bayes-Genis, 2009), hypertension (Carrick et al., 2018), and diabetic cardiomyopathy (Kibel et al., 2017; Sara et al., 2019). Impaired microvascular blood flow may also be a major contributor to HFpEF (Srivaratharajah et al., 2016; Dryer et al., 2018), for which there is no effective treatment at present. Cumulative human and animal data suggest that impaired microvascular blood flow may be due to low TH function in affected tissues. Indeed, microvascular blood flow is impaired in non-cardiac patients with subclinical hypothyroidism (Baycan et al., 2007). Recent rat studies from our group have shown that in hypertension (Carrillo-Sepulveda et al., 2019) and ischemic HF (Zhang et al., 2018), aortic rings are less responsive to the vasodilator acetylcholine. Pre-incubation with T3 largely restores vessel responsiveness toward normal. When considering the ubiquitous nature of fetal gene re-expression in heart diseases leading to HF and the likelihood that impaired microvascular blood flow is due to low TH function, restoration of normal TH function may have significant consequences for a broad range of cardiac patients. Additional benefits include improved LV contraction/relaxation, reduced fibrosis, reduced inflammation, and reduced cardiac morbidity and mortality. We are optimistic that these predictions based on scientific observations will form the basis for more clinical trials to test the potential efficacy of TH treatment in patients with cardiovascular diseases. Coronary blood flow measurements are not listed in the protocol (NCT04111536) for the just initiated NIH-funded T3 treatment of HFpEF patients led by Anne Cappola at U Penn. We think this should be a consideration.

### Study Limitations

It is difficult to distinguish whether effects of T3 treatment on BNP are direct or secondary. Indeed, changes in gene function, cardiac function, and remodeling are so intimately linked that cause-effect relationships are generally impossible to discern. In both models, T3 induced LV unloading and, by inference, reduced stretch activation of BNP as evidenced by reduced LVEDP.

Results from this study confirm a strong inverse correlation between BNP and T3 in heart diseases and suggest an important new use for this commonly used serum biomarker. We have not yet investigated the mechanism by which T3 may lead to downregulation of BNP. Potential mechanisms of negative regulation of genes by T3 are poorly understood. A previous report indicated the presence of a TRE distal from the promoter region of the human BNP gene (-1000; LaPointe, 2005). This may be a fruitful direction for future studies. Regarding the best studied gene negatively regulated by T3, β-MHC, a unique situation involves microRNAs localized within the α-MHC gene (Callis et al., 2009). It will likely take many years to fully understand the molecular mechanism of this inverse relationship between BNP and T3; however, the present report should stimulate more work in this area.

## Conclusion

Published clinical and animal studies to date suggest a strong inverse relationship between serum BNP and T3 in HF. Rat studies reported here confirm this relationship while also showing that when T3 treatment reduces serum BNP, this is associated with reversal of cardiac fetal genes and improvement in cardiac function. We believe that BNP may be a useful serum biomarker to guide T3 treatment in HF.

## Data Availability Statement

The raw data supporting the conclusions of this article will be made available by the authors, without undue reservation.

## Ethics Statement

The animal study was reviewed and approved by New York Institute of Technology Institutional Animal Care and Use Committee.

## Author Contributions

AG and KO contributed to the study design and oversight, the experimental methods development, study execution, the data collection, the analysis, and the interpretation. KW, AG, and KO wrote the manuscript. KW, AS, NG, SA, KZ, YZ, and BA contributed to the experiment execution, the data collection, the analysis, and the interpretation Y-DT contributed to the study design and interpretation. All authors edited and approved the final version of the manuscript.

## Conflict of Interest

The authors declare that the research was conducted in the absence of any commercial or financial relationships that could be construed as a potential conflict of interest.

## References

[B1] Amin A. Chitsazan M. Taghavi S. Ardeshiri M. (2015). Effects of triiodothyronine replacement therapy in patients with chronic stable heart failure and low-triiodothyronine syndrome: a randomized, double-blind, placebo-controlled study. *ESC Heart Fail* 2 5–11. 10.1002/ehf2.12025 28834641 PMC5746964

[B2] Balion C. Santaguida P. L. Hill S. Worster A. McQueen M. Oremus M. (2006). Testing for BNP and NT-proBNP in the diagnosis and prognosis of heart failure. *Evid. Rep. Technol. Assess.* 142 1–14. PMC4781047 17764210

[B3] Baycan S. Erdogan D. Caliskan M. Pamuk B. O. Ciftci O. Gullu H. (2007). Coronary flow reserve is impaired in subclinical hypothyroidism. *Clin. Cardiol.* 30 562–566. 10.1002/clc.20132 18000961 PMC6653161

[B4] Blaauw E. van Nieuwenhoven F. A. Willemsen P. Delhaas T. Prinzen F. W. Snoeckx L. H. (2010). Stretch-induced hypertrophy of isolated adult rabbit cardiomyocytes. *Am. J. Physiol. Heart Circ. Physiol.* 299 H780–H787. 20639217 10.1152/ajpheart.00822.2009

[B5] Brozaitiene J. Mickuviene N. Podlipskyte A. Burkauskas J. Bunevicius R. (2016). Relationship and prognostic importance of thyroid hormone and N-terminal pro-B-Type natriuretic peptide for patients after acute coronary syndromes: a longitudinal observational study. *BMC Cardiovasc. Disord.* 16:45. 10.1186/s12872-016-0226-2 26892923 PMC4757967

[B6] Bunevicius R. Varoneckas G. Prange A. J. Hinderliter A. L. Gintauskiene V. Girdler S. S. (2006). Depression and thyroid axis function in coronary artery disease: impact of cardiac impairment and gender. *Clin. Cardiol.* 29 170–174. 10.1002/clc.4960290409 16649727 PMC6654096

[B7] Callis T. E. Pandya K. Seok H. Y. Tang R. H. Tatsuguchi M. Huang Z. P. (2009). MicroRNA-208a is a regulator of cardiac hypertrophy and conduction in mice. *J. Clin. Invest.* 119 2772–2786. 10.1172/jci36154 19726871 PMC2735902

[B8] Cameron V. A. Ellmers L. J. (2003). Minireview: natriuretic peptides during development of the fetal heart and circulation. *Endocrinology* 144 2191–2194. 10.1210/en.2003-0127 12746273

[B9] Carrick D. Haig C. Maznyczka A. M. Carberry J. Mangion K. Ahmed N. (2018). Hypertension, microvascular pathology, and prognosis after an acute myocardial infarction. *Hypertension* 72 720–730. 10.1161/hypertensionaha.117.10786 30012869 PMC6080885

[B10] Carrillo-Sepulveda M. A. Panackal A. Maracheril R. Maddie N. Patel M. N. Ojamaa K. (2019). Triiodothyronine reduces vascular dysfunction associated with hypertension by attenuating PKG/VASP signaling. *J. Pharmacol. Exp. Ther.* 371 88–94. 10.1124/jpet.119.260471 31300610

[B11] Chen Y. F. Weltman N. Y. Li X. Youmans S. Krause D. Gerdes A. M. (2013). Improvement of left ventricular remodeling after myocardial infarction with eight weeks L-thyroxine treatment in rats. *J. Transl. Med.* 11:40. 10.1186/1479-5876-11-40 23409791 PMC3576349

[B12] Chuang C. P. Jong Y. S. Wu C. Y. Lo H. M. (2014). Impact of triiodothyronine and N-terminal pro-B-type natriuretic peptide on the long-term survival of critically ill patients with acute heart failure. *Am. J. Cardiol.* 113 845–850. 10.1016/j.amjcard.2013.11.039 24406111

[B13] Daubert M. A. Adams K. Yow E. Barnhart H. X. Douglas P. S. Rimmer S. (2019). NT-proBNP goal achievement is associated with significant reverse remodeling and improved clinical outcomes in HFrEF. *JACC Heart Fail* 7 158–168. 10.1016/j.jchf.2018.10.014 30611722

[B14] Di Angelantonio E. Chowdhury R. Sarwar N. Ray K. K. Gobin R. Saleheen D. (2009). B-type natriuretic peptides and cardiovascular risk: systematic review and meta-analysis of 40 prospective studies. *Circulation* 120 2177–2187. 10.1161/circulationaha.109.884866 19917883

[B15] Dryer K. Gajjar M. Narang N. Lee M. Paul J. Shah A. P. (2018). Coronary microvascular dysfunction in patients with heart failure with preserved ejection fraction. *Am. J. Physiol. Heart Circ. Physiol.* 314 H1033–H1042. 29424571 10.1152/ajpheart.00680.2017 PMC6008137

[B16] Du J. B. Da C. H. Zhao Y. Guo Y. Guo G. Ju T. F. (2012). The role of brain natriuretic peptide and serum triiodothyronine in the diagnosis and prognosis of chronic heart failure. *Acta Cardiol.* 67 291–296. 10.1080/ac.67.3.2160717 22870736

[B17] Escobar del Rey F. Ruiz de Ona C. Bernal J. Obregon M. J. de Escobar G. M. (1989). Generalized deficiency of 3,5,3’-triiodo-L-thyronine (T3) in tissues from rats on a low iodine intake, despite normal circulating T3 levels. *Acta Endocrinol.* 120 490–498. 10.1530/acta.0.1200490 2718701

[B18] Escobar-Morreale H. F. Botella-Carretero J. I. Escobar del Rey F. Morreale de Escobar G. (2005). REVIEW: treatment of hypothyroidism with combinations of levothyroxine plus liothyronine. *J. Clin. Endocrinol. Metab.* 90 4946–4954. 10.1210/jc.2005-0184 15928247

[B19] Gil-Cayuela C. Ortega A. Tarazon E. Martinez-Dolz L. Cinca J. Gonzalez-Juanatey J. R. (2018). Myocardium of patients with dilated cardiomyopathy presents altered expression of genes involved in thyroid hormone biosynthesis. *PLoS One* 13:e0190987. 10.1371/journal.pone.0109987 29320567 PMC5761948

[B20] Gil-Cayuela C. Rosello L. E. Tarazon E. Ortega A. Sandoval J. Martinez-Dolz L. (2017). Thyroid hormone biosynthesis machinery is altered in the ischemic myocardium: an epigenomic study. *Int. J. Cardiol.* 243 27–33. 10.1016/j.ijcard.2017.05.042 28526543

[B21] Guidelines for the Care and Use of Laboratory Animals. (2011). *National Research Council (US) Committee for the update of the guidelines for the care and use of laboratory animals. 8th Edn.* Washington, DC: National Academics Press (US).

[B22] Hajje G. Saliba Y. Itani T. Moubarak M. Aftimos G. Fares N. (2014). Hypothyroidism and its rapid correction alter cardiac remodeling. *PLoS One* 9:e109753. 10.1371/journal.pone.0109753 25333636 PMC4198123

[B23] Holmager P. Schmidt U. Mark P. Andersen U. Dominguez H. Raymond I. (2015). Long-term L-Triiodothyronine (T3) treatment in stable systolic heart failure patients: a randomised, double-blind, cross-over, placebo-controlled intervention study. *Clin. Endocrinol.* 83 931–937. 10.1111/cen.12648 25359424

[B24] Hunt P. J. Yandle T. G. Nicholls M. G. Richards A. M. Espiner E. A. (1995). The amino-terminal portion of pro-brain natriuretic peptide (Pro-BNP) circulates in human plasma. *Biochem. Biophys. Res. Commun.* 214 1175–1183. 10.1006/bbrc.1995.2410 7575527

[B25] Kibel A. Selthofer-Relatic K. Drenjancevic I. Bacun T. Bosnjak I. Kibel D. (2017). Coronary microvascular dysfunction in diabetes mellitus. *J. Int. Med. Res.* 45 1901–1929. 10.1177/0300060516675504 28643578 PMC5805190

[B26] LaPointe M. C. (2005). Molecular regulation of the brain natriuretic peptide gene. *Peptides* 26 944–956. 10.1016/j.peptides.2004.08.028 15911064

[B27] Liang F. Webb P. Marimuthu A. Zhang S. Gardner D. G. (2003). Triiodothyronine increases brain natriuretic peptide (BNP) gene transcription and amplifies endothelin-dependent BNP gene transcription and hypertrophy in neonatal rat ventricular myocytes. *J. Biol. Chem.* 278 15073–15083. 10.1074/jbc.m207593200 12562779

[B28] Liu Y. Redetzke R. A. Said S. Pottala J. V. de Escobar G. M. Gerdes A. M. (2008). Serum thyroid hormone levels may not accurately reflect thyroid tissue levels and cardiac function in mild hypothyroidism. *Am. J. Physiol. Heart Circ. Physiol.* 294 H2137–H2143. 18310509 10.1152/ajpheart.01379.2007

[B29] Man J. Barnett P. Christoffels V. M. (2018). Structure and function of the Nppa-Nppb cluster locus during heart development and disease. *Cell Mol. Life Sci.* 75 1435–1444. 10.1007/s00018-017-2737-0 29302701 PMC5852170

[B30] Mayer O. Jr. Simon J. Cech J. Rosolova H. Hrbkova J. Pikner R. (2008). Even mild changes in free thyroxine could influence the degree of heart failure measured by its biological surrogates. *Physiol. Res.* 57 525–529. 17705668 10.33549/physiolres.931172

[B31] Mitchell J. E. Hellkamp A. S. Mark D. B. Anderson J. Johnson G. W. Poole J. E. (2013). Thyroid function in heart failure and impact on mortality. *JACC Heart Fail* 1 48–55. 10.1016/j.jchf.2012.10.004 24159562 PMC3803999

[B32] Mygind N. D. Michelsen M. M. Pena A. Qayyum A. A. Frestad D. Christensen T. E. (2016). Coronary microvascular function and myocardial fibrosis in women with angina pectoris and no obstructive coronary artery disease: the iPOWER study. *J. Cardiovasc. Magn. Reson.* 18:76. 10.1186/s12968-016-0295-5 PMC5096323 27809867

[B33] Ozmen B. Ozmen D. Parildar Z. Mutaf I. Bayindir O. (2007). Serum N-terminal-pro-B-type natriuretic peptide (NT-pro-BNP) levels in hyperthyroidism and hypothyroidism. *Endocr. Res.* 32 1–8. 10.1080/07435800701670047 18271501

[B34] Pfister R. Strack N. Wielckens K. Malchau G. Erdmann E. Schneider C. A. (2010). The relationship and prognostic impact of low-T3 syndrome and NT-pro-BNP in cardiovascular patients. *Int. J. Cardiol.* 144 187–190. 10.1016/j.ijcard.2009.03.137 19423177

[B35] Pinelli M. Bindi M. Cassetti G. Moroni F. Pandolfo C. Rosada J. (2007). Relationship between low T3 syndrome and NT-proBNP levels in non-cardiac patients. *Acta Cardiol.* 62 19–24. 10.2143/ac.62.1.2019366 17375888

[B36] Pingitore A. Galli E. Barison A. Iervasi A. Scarlattini M. Nucci D. (2008). Acute effects of triiodothyronine (T3) replacement therapy in patients with chronic heart failure and low-T3 syndrome: a randomized, placebo-controlled study. *J. Clin. Endocrinol. Metab.* 93 1351–1358. 10.1210/jc.2007-2210 18171701

[B37] Pol C. J. Muller A. Zuidwijk M. J. van Deel E. D. Kaptein E. Saba A. (2011). Left-ventricular remodeling after myocardial infarction is associated with a cardiomyocyte-specific hypothyroid condition. *Endocrinology* 152 669–679. 10.1210/en.2010-0431 21159857

[B38] Rajagopalan V. Zhang Y. Ojamaa K. Chen Y. F. Pingitore A. Pol C. J. (2016). Safe Oral Triiodo-L-thyronine therapy protects from post-infarct cardiac dysfunction and arrhythmias without cardiovascular adverse effects. *PLoS One* 11:e0151413. 10.1371/journal.pone.0151413 26981865 PMC4794221

[B39] Roura S. Bayes-Genis A. (2009). Vascular dysfunction in idiopathic dilated cardiomyopathy. *Nat. Rev. Cardiol.* 6 590–598. 10.1038/nrcardio.2009.130 19636323

[B40] Saba A. Donzelli R. Colligiani D. Raffaelli A. Nannipieri M. Kusmic C. (2014). Quantification of thyroxine and 3,5,3’-triiodo-thyronine in human and animal hearts by a novel liquid chromatography-tandem mass spectrometry method. *Horm. Metab. Res.* 46 628–634. 10.1055/s-0034-1368717 24591048

[B41] Sara J. D. Taher R. Kolluri N. Vella A. Lerman L. O. Lerman A. (2019). Coronary microvascular dysfunction is associated with poor glycemic control amongst female diabetics with chest pain and non-obstructive coronary artery disease. *Cardiovasc. Diabetol.* 18:22. 10.1186/s12933-019-0833-1 PMC6393964 30819191

[B42] Sato Y. Yoshihisa A. Kimishima Y. Kiko T. Kanno Y. Yokokawa T. (2019). Low T3 syndrome is associated with high mortality in hospitalized patients with heart failure. *J. Card. Fail* 25 195–203. 10.1016/j.cardfail.2019.01.007 30682427

[B43] She J. Feng J. Deng Y. Sun L. Wu Y. Guo M. (2018). Correlation of triiodothyronine level with in-hospital cardiac function and long-term prognosis in patients with acute myocardial infarction. *Dis. Mark. 2018*:5236267. 10.1155/2018/5236267 PMC6304898 30627225

[B44] Srivaratharajah K. Coutinho T. deKemp R. Liu P. Haddad H. Stadnick E. (2016). Reduced myocardial flow in heart failure patients with preserved ejection fraction. *Circ. Heart Fail* 9:e002562. 10.1161/CIRCHEARTFAILURE.115.002562 27413034

[B45] Sudoh T. Kangawa K. Minamino N. Matsuo H. (1988). A new natriuretic peptide in porcine brain. *Nature* 332 78–81. 10.1038/332078a0 2964562

[B46] Tang Y. D. Kuzman J. A. Said S. Anderson B. E. Wang X. Gerdes A. M. (2005). Low thyroid function leads to cardiac atrophy with chamber dilatation, impaired myocardial blood flow, loss of arterioles, and severe systolic dysfunction. *Circulation* 112 3122–3130. 10.1161/circulationaha.105.572883 16275864

[B47] Thygesen K. Mair J. Mueller C. Huber K. Weber M. Plebani M. (2012). Recommendations for the use of natriuretic peptides in acute cardiac care: a position statement from the study group on biomarkers in cardiology of the ESC working group on acute cardiac care. *Eur. Heart J.* 33 2001–2006. 10.1093/eurheartj/ehq509 21292681

[B48] Tseng F. Y. Lin W. Y. Lin C. C. Lee L. T. Li T. C. Sung P. K. (2012). Subclinical hypothyroidism is associated with increased risk for all-cause and cardiovascular mortality in adults. *J. Am. Coll. Cardiol.* 60 730–737. 10.1016/j.jacc.2012.03.047 22726629

[B49] van den Heuvel A. F. van Veldhuisen D. J. van der Wall E. E. Blanksma P. K. Siebelink H. M. Vaalburg W. M. (2000). Regional myocardial blood flow reserve impairment and metabolic changes suggesting myocardial ischemia in patients with idiopathic dilated cardiomyopathy. *J. Am. Coll. Cardiol.* 35 19–28. 10.1016/s0735-1097(99)00499-4 10636254

[B50] Wang W. Guan H. Gerdes A. M. Iervasi G. Yang Y. Tang Y. D. (2015). Thyroid status, cardiac function, and mortality in patients with idiopathic dilated cardiomyopathy. *J. Clin. Endocrinol. Metab.* 100 3210–3218. 10.1210/jc.2014-4159 26052725

[B51] Wei T. Zeng C. Tian Y. Chen Q. Wang L. (2005). B-type natriuretic peptide in patients with clinical hyperthyroidism. *J. Endocrinol. Invest.* 28 8–11. 10.1007/bf03345522 15816364

[B52] Weltman N. Y. Ojamaa K. Savinova O. V. Chen Y. F. Schlenker E. H. Zucchi R. (2013). Restoration of cardiac tissue thyroid hormone status in experimental hypothyroidism: a dose-response study in female rats. *Endocrinology* 154 2542–2552. 10.1210/en.2012-2087 23594789 PMC3689280

[B53] Weltman N. Y. Ojamaa K. Schlenker E. H. Chen Y. F. Zucchi R. Saba A. (2014). Low-dose T(3) replacement restores depressed cardiac T(3) levels, preserves coronary microvasculature and attenuates cardiac dysfunction in experimental diabetes mellitus. *Mol. Med.* 20 302–312. 10.2119/molmed.2013.00040 24960246 PMC4153843

[B54] Weltman N. Y. Pol C. J. Zhang Y. Wang Y. Koder A. Raza S. (2015). Long-term physiological T3 supplementation in hypertensive heart disease in rats. *Am. J. Physiol. Heart Circ. Physiol.* 309 H1059–H1065. 26254335 10.1152/ajpheart.00431.2015 PMC4591362

[B55] Zhang K. Tang Y. D. Zhang Y. Ojamaa K. Li Y. Saini A. S. (2018). Comparison of therapeutic triiodothyronine versus metoprolol in the treatment of myocardial infarction in rats. *Thyroid* 28 799–810. 10.1089/thy.2017.0544 29580170 PMC5994663

