# Supplementary material for: BNP as a New Biomarker of Cardiac Thyroid Hormone Function
Source: Front Physiol. 2020 Jul 9;11:729. doi: 10.3389/fphys.2020.00729 (PMC7363952; doi:10.3389/fphys.2020.00729)
Supplement: Supplementary file 1 [file Table_1.DOCX]

| Supplemental Table. Cardiac echocardiographic recordings and morphometric measurements | | | | |  |
| --- | --- | --- | --- | --- | --- |
|  | **EU** | **PTU** | **PTU+3d T3** | **PTU+6d T3** | **PTU+14d T3** |
| BW, g | 256±26 | 209±16^a^ | 206±4^a^ | 209±26^a^ | 225±6 |
| HW, mg | 759±70 | 568±37^a^ | 637±24^a,b^ | 652±57^a,b^ | 794±13 |
| LVW, mg | 616±66 | 461±22^a^ | 521±10^a,b^ | 527±46^a,b^ | *nd* |
| LV/BW, mg/g | 2.4±0.13 | 2.2±0.11^a^ | 2.5±0.05^b^ | 2.5±0.13^b^ | *nd* |
| HW/BW, mg/g | 2.97±0.16 | 2.85±0.23 | 3.10±0.07 | 3.12±0.15 | 3.53±0.13^a,b,c,d^ |
| LVPWs, mm | 2.12±0.05 | 1.66±0.04^a^ | 2.02±0.11^b^ | 1.98±0.05^b^ | 2.05±0.01^b^ |
| LVIDs, mm | 3.45±0.27 | 4.618±0.37 | 3.57±0.64 | 3.49±0.39 | 3.48±0.37 |
| EF, % | 82±1 | 70±5^a^ | 82±5^b^ | 82±4^b^ | 85±4^b^ |
| AWTs, mm | 2.08±0.05 | 1.66±0.04^a^ | 2.02±0.15^b^ | 1.95±0.0^b^ | 2.14±0.12^b^ |
| LVPWd, mm | 1.23±0.0 | 1.03±0.0^a^ | 1.16±0.05^b^ | 1.16±0.05^b^ | 1.10±0.05^a^ |
| LVIDd, mm | 6.33±0.35 | 6.99±0.24 | 6.50±0.62^b^ | 6.43±0.17 | 6.91±0.39 |
| AWTd, mm | 1.23±0.0 | 1.03±0.0^a^ | 1.13±0.0^a,b^ | 1.13±0.0^a,b^ | 1.10±0.05^a,b^ |

EU, euthyroid; BW, body weight; HW, heart wt; LVW, LV wt; ejection fraction (EF); left ventricle posterior wall thickness in diastole (LVPWd) and systole (LVPWs); left ventricle internal diameter in diastole (LVIDd) and systole (LVIDs); left ventricle anterior wall thickness in diastole (AWTd) and systole (AWTs). Values are mean ± SD. Rats/group: EU, n=6-8; PTU, n=6-9; 3d T3, n=6; 6d T3, n=6; 14d T3, n=6-10. *nd* = not determined. Statistical analysis used one-way ANOVA with Tukey’s post-hoc multiple-group comparisons. ^a^ p< 0.05 vs control; ^b^p< 0.05 vs PTU; ^C^p< 0.05 vs PTU+3d T3; ^d^p< 0.05 vs PTU+ 6d T3.
